# A Novel Microcrystalline BAY-876 Formulation Achieves Long-Acting Antitumor Activity Against Aerobic Glycolysis and Proliferation of Hepatocellular Carcinoma

**DOI:** 10.3389/fonc.2021.783194

**Published:** 2021-11-18

**Authors:** Hua Yang, Mu-Zi-he Zhang, Hui-wei Sun, Yan-tao Chai, Xiaojuan Li, Qiyu Jiang, Jun Hou

**Affiliations:** ^1^ Department of Medical Oncology, Affiliated Hospital of Hebei University, Hebei Key Laboratory of Cancer Radiotherapy and Chemotherapy, Baoding, China; ^2^ Department of Pharmacy, Medical Security Center of PLA General Hospital, Beijing, China; ^3^ Department of Infectious Disease, Institute of Infectious Disease, The Fifth Medical Center of Chinese PLA General Hospital, Beijing, China

**Keywords:** advanced hepatocellular carcinoma, glucose transporter type 1, BAY-876, microcrystalline, aerobic glycolysis, epithelial-mesenchymal transition

## Abstract

BAY-876 is an effective antagonist of the Glucose transporter type 1 (GLUT1) receptor, a mediator of aerobic glycolysis, a biological process considered a hallmark of hepatocellular carcinoma (HCC) together with cell proliferation, drug-resistance, and metastasis. However, the clinical application of BAY-876 has faced many challenges. In the presence study, we describe the formulation of a novel microcrystalline BAY-876 formulation. A series of HCC tumor models were established to determine not only the sustained release of microcrystalline BAY-876, but also its long-acting antitumor activity. The clinical role of BAY-876 was confirmed by the increased expression of GLUT1, which was associated with the worse prognosis among advanced HCC patients. A single dose of injection of microcrystalline BAY-876 directly in the HCC tissue achieved sustained localized levels of Bay-876. Moreover, the single injection of microcrystalline BAY-876 in HCC tissues not only inhibited glucose uptake and prolonged proliferation of HCC cells, but also inhibited the expression of epithelial-mesenchymal transition (EMT)-related factors. Thus, the microcrystalline BAY-876 described in this study can directly achieve promising localized effects, given its limited diffusion to other tissues, thereby reducing the occurrence of potential side effects, and providing an additional option for advanced HCC treatment.

## Introduction

Despite the current progress of hepatocellular carcinoma (HCC)-related research, the overall prognosis of advanced HCC is still poor (1, 2). Increasing evidence has indicated that aberrant glucose metabolism (aerobic glycolysis/Warburg effect) is a hallmark of HCC and is active in the tumor microenvironment, tumor proliferation, drug-resistance, or epithelial-mesenchymal transition (EMT) (3–5). The liver is a pivotal organ responsible for the body’s energy metabolism and the excessive uptake of glucose by HCC cells is the key to abnormal metabolism, a mechanism which is mediated by glucose transporters (GLUT) (6–9). Therefore, it is valuable to identify new therapeutic agents for intervention in advanced HCC treatment targeting the GLUTs.

GLUT1 (Glucose transporter type 1) is the most common member of the GLUTs and is widely expressed in adult organs. Under stress factors such as hypoxia, low partial pressure of oxygen, and changes in osmotic pressure, cells can uptake a large amount of glucose through GLUT1 to meet the body’s energy metabolism requirements (10–12). Over-expression of GLUT1 has been identified during tumorigenesis and progression in several kinds of human malignancies, and is regulated by hypoxia, steroid hormones, growth hormones, insulin, and some oncogenes (13–15). At present, there have been few reports investigating GLUT1 inhibitors (16–20). Among these, the most specific, most representative and effective is BAY-876, which is water-insoluble yellow powder (21). BAY-876 has strong inhibitory activation on GLUT1, and the IC50 value in a cell-free system is low as 2 nmol/L (21). However, the clinical application of BAY-876 still faces many challenges. First, most patients with HCC in China have intolerable cirrhosis, the patient’s gastrointestinal function is insufficient/impaired, which results in such patients having poor absorption of oral drugs (22, 23). Secondly, oral administration or intravenous infusion as the route of administration will cause the drug to be distributed systemically, which will not only interferes with the physical uptake of glucose of the human body, but also results in negligible dose of BAY-876 at the lesion site of HCC (24–27). Therefore, the aim of this study was to establish a long-acting and sustained-release formulation of BAY-876 to achieve precise dose administration, and exert targeted anti-tumor activity at HCC lesions without affecting normal tissues and organs. The BAY-876 pure powder was prepared as a microcrystalline formulation characterized by a larger particle size of the drug crystals (of micron-magnitude). Based on the insoluble properties of BAY-876, the microcrystalline formulation is injected into the tumor tissue directly. The tumor cells in the tissue slowly dissolve BAY-876 crystals to achieve sustained and long-term drug release. At the same time, as the size of the microcrystals is larger and the release is slower.

## Materials and Methods

### Cell Culture and Reagents

L-02, a normal immortalized liver-derived cell line, grows adherently and presents epithelial cell-like characteristics andliver-derived HCC cell lines: HepG2, MHCC97-H (a highly invasive HCC cell line) and MHCC97-L (a minimally invasive cell line) (28–30) were used for this study. The fetal bovine serum (FBS) used in the cell cultures was purchased from Hyclone, USA; and cell cultures, DMEM cell culture medium was purchase from Invitrogen together with trypsin for cell digestion. The 96-well plate for cell cultures, 60-mm and 90-mm petri dishes were purchased from Corning Corporation of the United States. The GLUT1 antagonist BAY-876 was purchased from Selleck, USA (product number: S8452). Other reagents including the dimethyl sulfoxide, Tween 80, and polyethylene glycol 400 used in the study were all analytical pure grade and were gifts from Dr. Tao Wang in Beijing Institute of Pharmacology.

### Patient Data and Clinical Samples

The patient data in this study and the clinical samples for RNA extraction and reverse transcription were provided by the research group of Feng and Shao et al., 2018 (30, 31). The criteria for patient enrollment and clinical characteristics of patients have been described in detail previously (30, 31). A total of 52 patient samples were used for this study. For the extraction of total RNA: a total of 1×10^5^ cells were suspended in PBS, and RNA extraction was performed according to the reagent instructions provided by QIAGEN. The final elution volume was 80 μL. The reverse transcription was performed according to the manufacturer’s instructions, using random primers as described in **Supplemental Tables 1–3**. The expression level of GLUT1 was detected by qPCR in HCC tumor tissues, and patients were divided into two groups according to the median of its expression: GLUT-1 high group and GLUT1-low group. Using the median and 95% confidence interval of OS and TTP to reveal the prognosis of patients. The primers used for analysis of gene expression were the following: GLUT1, forward primer 5’-TTGCA GGCTTCTCCAACTGGAC-3’, reverse primer 5’-CAGAACCAGGAGCACAGTGA AG-3’; β-Actin, forward primer 5’-ACCGCACACAGCAAGGCGAT-3’, reverse primer 5’-CGATTGAGG GCTCCTAGCGGTT-3’; E-cadherin, forward primer 5’-AAGGCACGCCTGTCGAAGCA-3’, reverse primer 5’-ACGTTGTCCCGGGTGTCATCCT-3’; N-cadherin, forward primer 5’-TGCGCGTGAAGGTTTGCCAGT-3’, reverse primer 5’-TGGCGTTCTTTATCC CGGCGT-3’; Vimentin, forward primer 5’-ACCGCACACAGCAAGGCGAT-3’, reverse primer 5’-CGATTGAG GGCTCCTAGCGGTT-3’; and (6) β-Actin forward primer 5’-ACCGCACACAGCAAGGCGAT-3’; reverse primer 5’-CGATTGAGGGCTCCTAGCGGTT-3’. In the PCR, the primers were diluted to a concentration of 10 μmol/L, and the qPCR amplification was prepared according to the instructions provided by the KAPA Reverse Transcription Reagent (**Supplemental Tables 1–3**). The ABI 7500 Real-time PCR instrument was used for gene amplification and analysis. β-Actin was used as the internal housekeeping control gene.

Moreover, the GLUT1 related data were also obtained from the databases to have a better overview of GLUT1 expression in a larger cohort of HCC patients (32–34). By using an online tool (http://gepia2.cancer-pku.cn/), the GLUT-1’s expression related data were obtained from the TCGA database or the GTEx database. The results were shown as the scatter-plot images of GLUT1 expression levels in HCC and non-tumor tissues or the survival curve of patients with GLUT1 high expression group and GLUT1 low expression group.

### Western Blot

HCC cells were cultured and transfected with plasmids encoding the small interfering RNA (siRNA) targeting GLUT1 (Control siRNA or GLUT1 siRNA) or treated with agents. The cells were digested with trypsin, resuspend in PBS, and then incubated in RIPA lysis buffer with protease inhibitors. The suspensions were vortexed to lyse cells under shaking conditions. Next, cell samples were fixed with SDS loading buffer for cleavage of proteins under boiling. SDS-PAGE was performed according to the standard protocol and the protein bands in the gel were transferred onto a PVDF membrane. Next, the membrane was blocked using 5% BSA (bovine serum albumin). The membrane was incubated and exposed to the primary antibodies at appropriate dilutions (GLUT1, 1:1000; β-Actin, 1:5000; the Abcam Corporation, Cambridge, UK) and to the secondary antibody (the IgG conjugated with horseradish Peroxidase [HRP], 1:5000 dilution, the Abcam Corporation, Cambridge, UK). Chemiluminescence reagents were used and then X-ray films were used for compression and development.

### Detection of MHCC97-H Cells by MTT

The HCC cell lines (MHCC97-H or HepG2) were transfected with the indicated plasmids and seeded into 96-well plates (at a seeding density of 2000 cells/well). Then, 50 μL of the prepared MTT solution (5 mg/mL MTT in serum-free DMEM) was added into the well at a series of time points. After a 5-hour incubation, the medium was discarded and 200 μL DMSO was added to each well, and shaken. A 150 μL volume of the above was added into a new 96-well plate, and the OD (absorbance) value at 490nm was read using a microplate reader (35–37).. Based on the fact that MTT is a quantification of cellular metabolic rate, to claim that GLUT1 silencing reduces proliferation, the cell counts at assay end point were further examined by the blood plate counter. The cells were harvested from the 96-well plates by the trypsin and the cell counts were examined.

### Formulation of BAY-876 Formulations

A 1.5 g amount of BAY-876 drug amorphous powder was weighed and suspended in a physiological saline solution containing a 6.25% (V/V) concentration of Tween 80. The mixture was stirred gently to ensure complete mixing of the drug and solvent. Next, the drug-solvent mixture was added to the working chamber of a high-speed shearing device, to pre-disperse the sample at 10,000 rpm for 10 minutes; when the pressure reached 50 MPa, the device was set to pre-grind for 5 minutes to obtain a drug suspension. The drug suspension was then added to the grinding kettle of the wet mill, and starting from the lowest grinding pressure, the pressure was gradually increased by 10 MPa every 5 minutes of grinding. When the grinding pressure finally reached 50 MPa, the device continued grinding for 5 minutes to obtain the microcrystalline formulation of BAY-876. The drug loading amount of the microcrystals prepared according to this method was 30 mg/mL. As a control, a BAY-876 solution was prepared in accordance with previous studies (38–41). The BAY-876 solution was named as BAY-Sol and the microcrystalline formulation of BAY-876 was named as BAY-Micro in the next steps’ experiments.

### Immunodeficiency Nude Mice Subcutaneous Tumor Formation Experiment

MHCC97-H cells were cultured and injected into nude mice to establish a subcutaneous xenograft tumor model. BalB/c immune-deficient (absence of thymus) mice were purchased from Si-bei-fu Corporation, Beijing, China. The handling of animals and relative experiments were approved by the animal Ethics Committee of the Hebei Key Laboratory of the Cancer Radiotherapy and Chemotherapy or The Fifth Medical Center of Chinese PLA General Hospital, Beijing, China. After 2–3 weeks of injection, the length and width of the nude mice’s subcutaneous tumors were measured. and the tumor volume was calculated as follows: tumor volume = subcutaneous tumor length × width × width/2, when the volume of the subcutaneous tumor reached approximately 1000-1200 mm^3^, the construction of the immunodeficiency nude mouse subcutaneous tumor model was completed (42–46). The Warburg effect related factors or EMT related factors in the were examined following the methods descripted by Yin et al., 2019 (47).

Among the subcutaneous tumor formation experiment, to confirm the inhibitory effect of BAY-876 on HCC cells’ glucose uptake, MHCC97-H cells were cultured and injected into nude mice to form the subcutaneous tumor tissues. After MHCC97-H cells form tumor tissues in the subcutaneous position of nude mice, 5 mg/kg BAY-876 was given by oral administration every day (once a day for two days). Then, the mice were analyzed by the micro-PET live imaging of small animals (nude mice),and the tumor tissues were collected for the biochemical analysis. For the antitumor activation experiments of Bay-876 formulations, MHCC97-H cells were cultured and injected into nude mice to form the subcutaneous tumor tissues. After MHCC97-H cells form tumor tissues, the formulations of BAY-876, the BAY-876 solution (BAY876-Sol) or microcrystalline formulation of BAY-876 (BAY876-Micro) was directly injected into the tumor tissues. The body-weights or the liver-weights of nude mice were examined.

### Pharmacokinetics of the Antitumor Activity by the BAY-876 Formulation

The pharmacokinetic properties of Bay-876 were determined in subcutaneous tumor model following the methods described previously (21, 42–47). Briefly, the BAY-876 solution or microcrystalline formulation was injected into the subcutaneous tumors and the tumor tissues were harvested at the indicated time points. The amount of BAY-876 in the tumor tissues was measured by LC-MS/MS. The LC-MS/MS method consisted of the negative ion mode and was set for three specific ion pairs (m/z: 495.5>189.5; 495.5>379; 495.5>305.0), the cone voltage was 50V; the collision energy was set to 30. To perform High Performance Liquid Chromatography (HPLC), a C18 chromatographic column was used (C18 LC-MS), with the following parameters: mobile phase (A liquid, 95% CAN; B liquid, 5% H_2_O; 0.1% formic acid; gradient, 0–4 min [2%A–80% A], 4–4.1 min [80% A–100% A], 4.1–6.0 min [100% A–100% A], 6.0–6.1 min [100% A–2% A], 6.1–10.0 min [2% A–2% A], and liquid phase flow rate 0.25 mL/min) (**Supplemental Table 4**). The mass spectrometry conditions were the following: cone voltage 50 V; source temperature 500°C; nebulization gas 8; rolling curtain gas 10; collision gas 6; and a scan time of 150 msec.

To evaluate the antitumor effect of BAY-876 formulations, the BAY-876 solution or microcrystalline formulation was injected into the subcutaneous tumors in nude mice or the intrahepatic lesions. The MHCC97-H cells were first cultured and then injected into nude mice to form the tumor tissues. The subcutaneous tumor tissues were collected and prepared as micro-blocks. The micro-blocks of tumor tissues were transplanted in the liver organs of immunodeficient rats to form the intrahepatic lesions. The same amount of a single dose of solvent control, BAY-876 solution or BAY-876 microcrystalline was directly injected into the subcutaneous tumor tissues or the intrahepatic lesions. The antitumor effect of these formulations was measured by determining tumor volumes, tumor weights, or using microPET. The aerobic glycolysis of the tumor tissues were measured following the methods described by Yin et al. (42). The body-weights or the liver-weights of Immunodeficiency rat were examined (24–27).

### Statistical Analysis

Significant differences among variables was performed using independent sample t-test using SPSS 24.0 software, while one-way analysis of variance was used to compare the mean ± standard deviation and to calculate the p-value.

## Results

### High GLUT1 Levels Were Correlated With Poor Prognosis of Advanced HCC Patients

**Figure 1:**
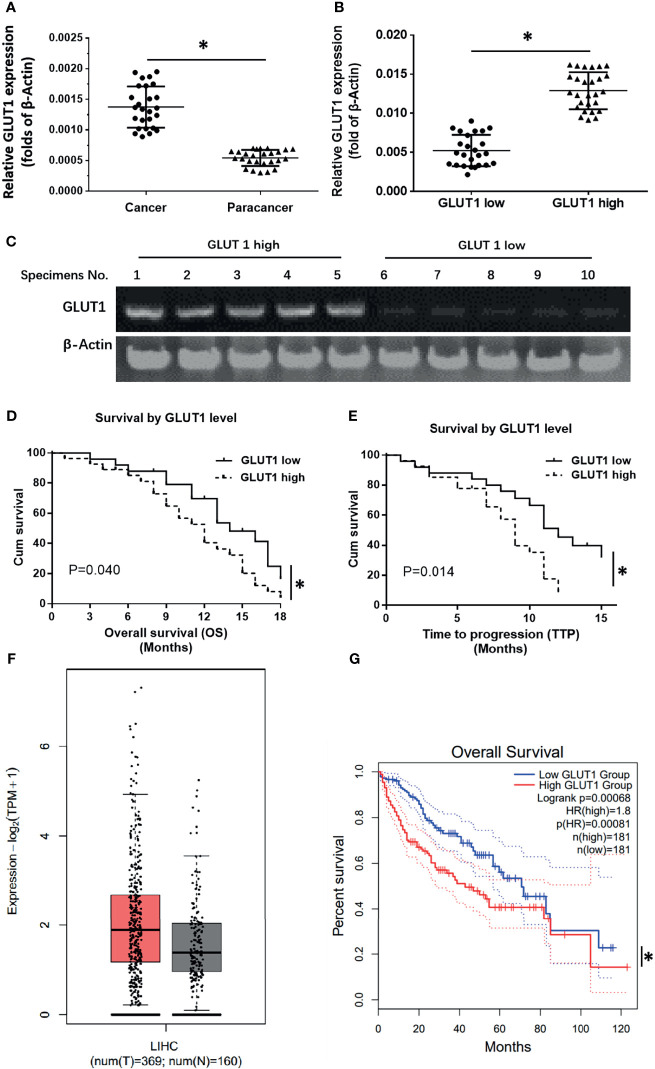
The clinical significance of GLUT1 in advanced HCC. **(A)** The endogenous expression of GLUT1 in HCC or the para-tumor non-tumor tissues was examined by qPCR and shown as scatter-plot images. **(B)** The endogenous expression of GLUT1 in HCC tissues was examined by qPCR and is shown as the scatter-plot images. **(C)** The patients were divided into two groups (the GLUT1 high expression group and the GLUT1 low expression group) and the representative GLUT1 DNA electropherograms are shown. **(D, E)** Survival outcomes (OS and TTP) of the two patient groups. **(F)** the GLUT1 expression in a larger cohort of HCC patients from database (the HCC and non-tumor tissues); The left group of the scatter-plot indicated the HCC groups (T, tumor) **(F)** and the right group of the scatter-plot indicated the paired non-tumor groups (N, non-tumor) **(F)**. **(G)** the prognosis of patients with high GLUT1 or low GLUT1 in a larger cohort of HCC patients from database. *P<0.05. LIHC, liver hepatocellular carcinoma.

To explore therapeutic strategies targeting GLUT1 the clinical role of GLUT1 in advanced HCC was examined. As shown in **Figure 1**, the expression of GLUT1 was much higher in HCC clinical specimens compared with the non-tumor/para-tumor tissues (**Figure 1A**). The clinical samples were divided into the GLUT1 high expression group (GLUT-1-high) and GLUT1 low expression group (GLUT-1-low) (**Figures 1B, C**) according to the median value of expression level of GLUT1. As shown in **Figures 1D, E**, the overall survival (OS) or the time to progression (TTP) of patients belonging to the GLUT-1-high group was much shorter compared to patients in the GLUT-1-low group. The median, 95% confidence interval (CI), PR (partial remission), CR (complete remission), and SD (stable disease) data and the P-values in the two groups of patients are shown in the **Table 1**. Next, the GLUT1 expression in a larger cohort of HCC patients was examined by using the data from TCGA or GTEx. As shown in **Figure 1F**, the expression of GLUT1 is much higher in HCC groups (n=369) than that in the non-tumor groups (n=160). The overall survival of HCC patients with high level of GLUT1 (high GLUT1 group, n=181) was worse compared with the HCC patients with low level of GLUT1 (low GLUT1 group, n=181) (**Figure 1G**).

**Table 1:** The relationship between the expression level of GLUT1 and the prognosis of patients with advanced hepatocellular carcinoma receiving sorafenib treatment.

	GLUT1 mRNA expression		P value
	High (n = 27)	Low (n = 25)	
TTP	9.0	12.0	0.0142
	7.9-10.1 (M)	9.6-14.4 (M)	
OS	12.0	14.0	0.0395
	9.6-14.4 (M)	10.2-17.8 (M)	
Overall response rate (CR + PR)	1 (3.7%)	3 (12.0%)	
Disease control rate (CR+PR+SD)	3 (11.1%)	10 (40.0%)	

TTP, time to progress; OS, overall survival; PR, partial remission; CR, complete remission; SD, stable of disease; M, months.

**Figure 2:**
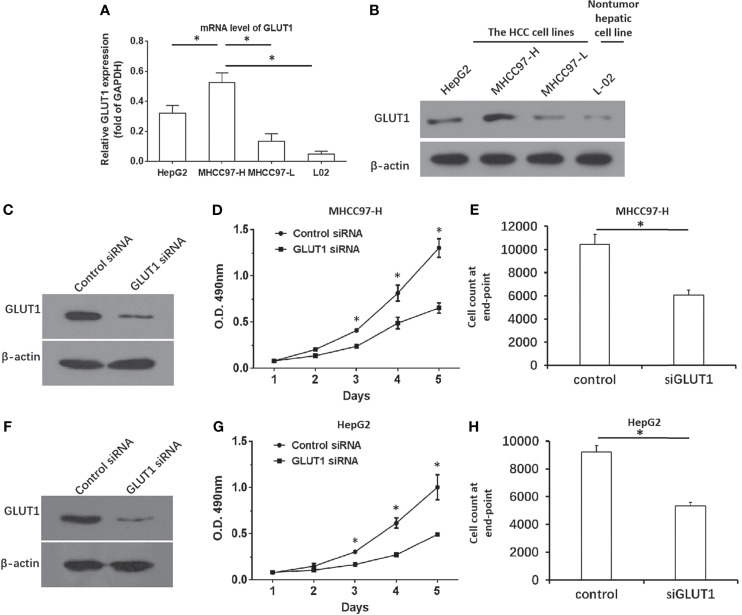
GLUT1 is a positive regulator of HCC cells. **(A, B)** The endogenous expression of GLUT1 in the hepatic cell lines was examined by qPCR **(A)** and western blot **(B)**. The results were shown as a histogram **(A)** and images of western bolt **(B)**. The MHCC97-H **(C–E)** or HepG2 **(F–H)** cells were transfected with control or siGLUT1. The protein levels of GLUT1 was examined by western blot and shown in **(C, F)**, while the proliferation of HCC cells was examined by the MTT and shown as the line chart in panels **(D, G)** The cell number at the end point was shown **(E, H)**. *P<0.05.

To confirm the roles for GLUT1 in HCC, the expression of GLUT1 was first examined in hepatic cell lines. As shown in **Figures 2A, B**, the endogenous expression of GLUT1 was much higher in MHCC97-H or HepG2 cells compared with the non-tumor L-02 cells or the minimally aggressive MHCC97-L cells. Moreover, the siRNA of GLUT1 was transfected to knockdown the expression of GLUT1 in MHCC97-H (**Figures 2C–E**) or HepG2 cells (**Figures 2F–H**). As shown in **Figures 2C–H**, knockdown of GLUT1 expression in HCC cells *via* the specific siRNA significantly inhibited the growth and proliferation of both cells lines. The results were shown as the images of western blot (**Figures 2B, C, F**), the cell-proliferation curves from MTT (**Figures 2D, G**), or the cell counts at the end point (**Figures 2E, H**). Thus, downregulation of GLUT1 could inhibit the proliferation of HCC cells and thus, it is meaningful to study anti-tumor strategies targeting GLUT1 in HCC.

### Effects of Different BAY-876 Formulations

**Figure 3:**
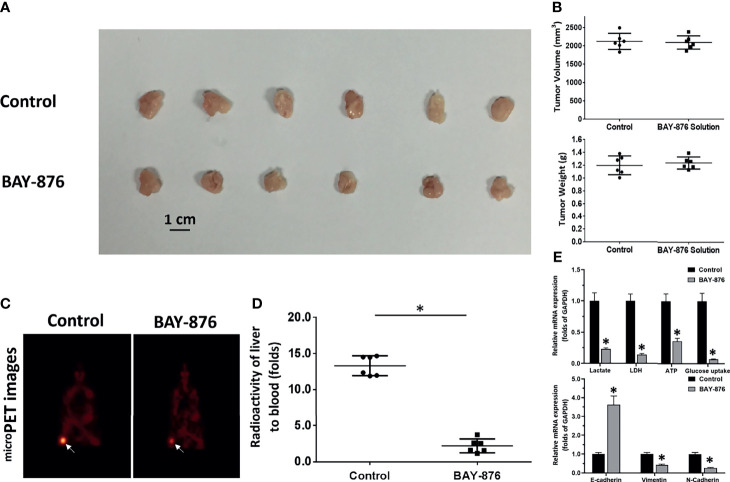
BAY-876 inhibited the Warburg effect of tumor tissues formed by MHCC97-H cells. The MHCC97-H cells were injected into the nude mice to form the subcutaneous tumor models. The mice received oral administration of BAY-876 for three days (once daily). After BAY-876 treatment, the mice were analyzed by microPET. The results are shown as images of subcutaneous tumor tissues **(A)**, tumor volumes or tumor weights **(B)**, microPET images **(C)**, and the quantitative analysis of microPET (the relative nuclide [18F-FDG] intensity [fold change of tumor to blood] by the Geiger counter) **(D)**, or the Warburg effect. EMT-related factors are shown in **(E)**. *P<0.05 *versus* control with BAY-876.

As mentioned above, targeting GLUT1 may have therapeutic effects on HCC cells. Therefore, the effects of BAY-876 on HCC cells was examined. The nude mice subcutaneous tumor model of MHCC97-H cells was established, and nude mice were treated by oral gavage daily for 3 days (**Figure 3**). As shown in **Figure 3**, MHCC97-H cells formed a subcutaneous tumors. The short-term treatment (only for two days) of BAY-876 for two days (5mg/kg dose of BAY-876, oral administration, one time for every day) did not affect the tumor volumes or tumor weights (**Figures 3A, B**). Nonetheless, oral administration of BAY-876 for two days significantly inhibited the glucose uptake of tumor tissues formed by MHCC97-H cells (**Figures 3C, D**) as indicated by the microPET images (**Figure 3C**). The intensity of 18F-FDG nuclide signals in the subcutaneous tumors of nude mice in the BAY-876 treatment group was significantly lower than that in the solvent control group (**Figure 3D**). Accordingly, reduced ATP levels, lactate production, glucose uptake, and LDH activation were in detected in tumor tissues upon BAY-876 treatment (**Figure 3E**). Treatment of BAY-876 also inhibited the EMT of HCC cells in tumor tissues (**Figure 3E**). These results further confirmed that treatment of BAY-876 inhibited the glucose uptake of HCC cells in tumor tissues.

**Figure 4:**
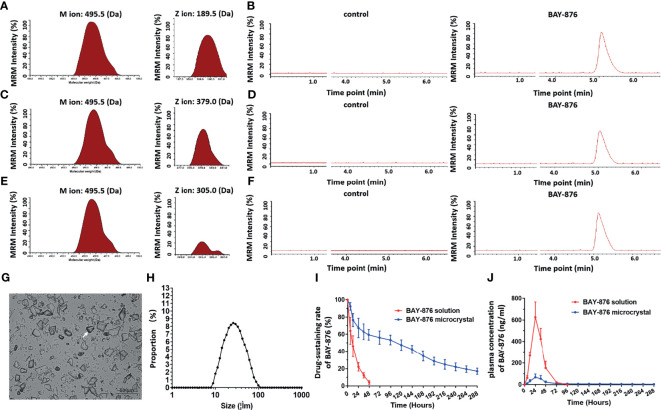
BAY-876 microcrystalline achieved long-sustaining release of BAY-876 in HCC tissues. **(A–F)** LC-MS/MS approach for the detection of BAY-876. **(G)** Images of BAY-876 microcrystalline structure. **(H)** Particle size distribution of Bay-876 microcrystalline. **(I)** *In vivo* drug-release rate curve of BAY-876 in HCC subcutaneous tumor tissues. **(J)** Blood concentration curve of BAY-876 in HCC subcutaneous tumor tissues injected with microcrystalline BAY-876.

Next, the microcrystalline formulation of BAY-876 was prepared. As shown in **Figures 4A–F**, BAY-876 concentrations were determined in negative ion mode *via* LC-MS/MS, by detecting the molecular weight of the parent ion of BAY-876 was 495.5 Da; three specific product ions (189.5 Da, 379.0 Da and 305 Da) of BAY-876 were discovered and identified. BAY-876 was detected only the BAY-876 group, while the blank control did not produce any signals. Next, the microcrystalline formulations of Bay-876 was prepared. The image of the microcrystalline BAY-876 formulation is shown as **Figure 4G**. The particle size distribution is shown as **Figure 4H**.

Furthermore, the microcrystalline BAY-876 formulation was injected directly into the subcutaneous tumor tissues to examine the *in vivo*-releasing capabilities of Bay-876 from the BAY-876 microcrystalline and the Bay-876 solution used as control. As shown in **Figures 4I, J**, after injection of the BAY-876 solution into tumor tissues, BAY-876 was almost completely metabolized and cleared from the tumor tissue within 24 hours. The blood concentration of BAY-876 quickly reached the maximum value (more than 700 ng/mL), and then the concentration dropped rapidly, and was no longer detected after 72 hours (**Figure 4J**). However, after injection of BAY-876 microcrystalline into tumor tissues, sustained blood levels of BAY-876 in tumor tissue were observed (**Figure 4I**), while the blood concentration of BAY-876 in the BAY-876 microcrystalline group was lower (approximately 100 ng/mL) and reached peak levels after about 24 hours and then rapidly and slowly dropped to very low levels (**Figure 4J**). Thus, the microcrystalline BAY-876 formulation achieved a prolonged and sustained BAY-876 levels in tumor tissues.

### BAY-876 Microcrystalline Achieved Long-Term Antitumor Activation in the Subcutaneous Tumor Model

**Figure 5:**
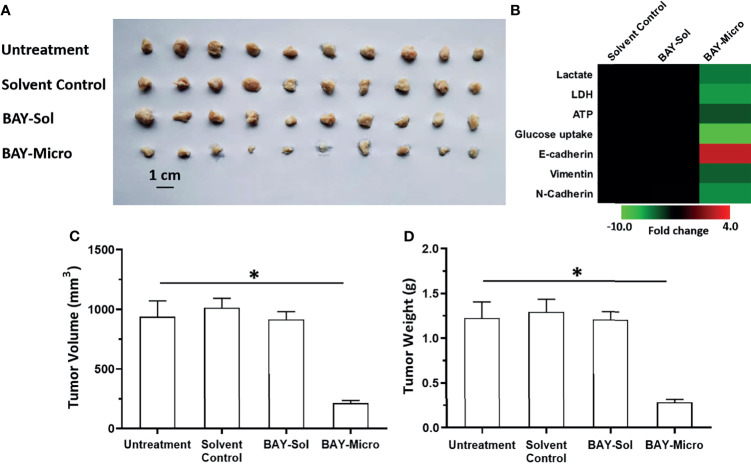
Long-acting anti-tumor activation of BAY-876 microcrystalline on the subcutaneous growth of MHCC97-H cells. The MHCC97-H cells were injected into the nude mice to form tumor tissues. The solvent control, BAY-Sol (BAY-876 solution), BAY-Micro (Bay-876 microcrystalline) was directly injected into the subcutaneous tumor tissues. The results are shown as representative images of subcutaneous tumor tissues **(A)**, the heat-map showing the effects of each treatment on the Warburg effect or on the EMT related factors **(B)**. **(C, D)** Tumor volumes and weights. *P<0.05.

The above results showed that compared with the BAY-876 solution, the microcrystalline BAY-876 formulation achieve prolonged and sustained levels of BAY-876 in tumor tissues. Therefore, the antitumor activity of the BAY-876 formulations was examined by using subcutaneous tumor models. As shown in **Figure 5**, a single intra-tumor injection of BAY-876 microcrystalline but not the solvent control could inhibit the subcutaneous growth of MHCC97-H cells. Accordingly, reduced ATP levels, lactate production, glucose uptake, and LDH activation were detected in tumor tissues treated with microcrystalline BAY-876 injection but not following treatment with Bay-876 solution or the solvent control (**Figure 5**). A single injection of the microcrystalline BAY-876 formulation but not the BAY-876 solution or solvent control also inhibited the EMT of HCC cells in tumor tissues (**Figure 5**). As shown in **Table 2**, the one dose of intra-tumor injection of BAY-876 solution or BAY-876 microcrystalline formulation (BAY-Micro) did not affect the body-weights or liver-weights of nude mice mentioned in **Figure 5**. Therefore, formulation of BAY-876 as microcrystals can achieve the long-term anti-tumor activity after the administration of a single dose and did not induce the significant toxicity, e.g. body-weights loss in animal.

**Table 2:** The body-weights and liver-weights of mice mentioned in **Figure 5**.

Groups	Body weight (g)	Liver (mg)
Untreatment	20.19 ± 4.62	620.00 ± 48.67
Solvent Control	21.36 ± 5.68	608.20 ± 51.66
BAY-Sol	20.67 ± 2.12	614.57 ± 20.29
BAY-Micro	19.76 ± 3.85	610.51 ± 38.69

BAY-Sol, BYA-876 solution; BAY-Micro, BAY-876 microcrystalline formulation.

### Long-Term Antitumor Activity of the Microcrystalline BAY-876 Formulation on the Intrahepatic Tumor Model in Immunodeficiency Rat

**Figure 6:**
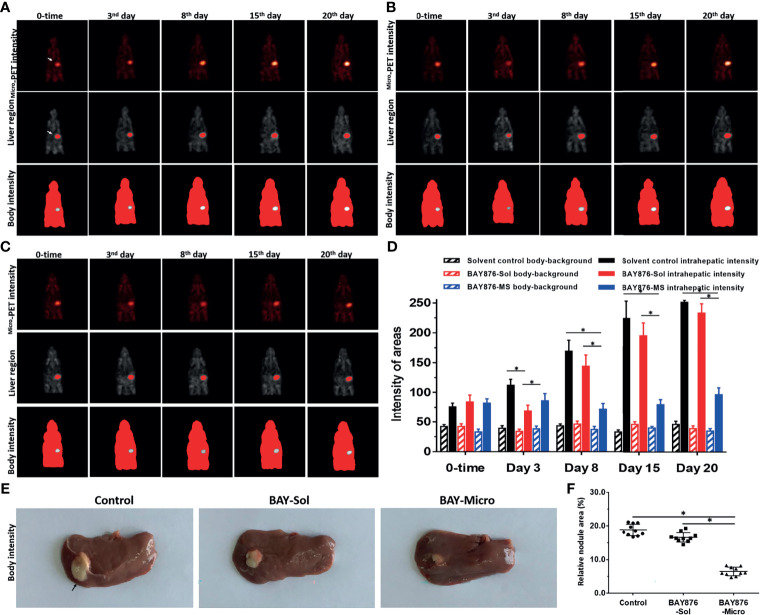
Effects of long-acting anti-tumor microcrystalline BAY-876 formulation on the intrahepatic of MHCC97-H cell tumor model. MHCC97-H cells were cultured to generate intrahepatic tumor lesions in the immunodeficient rat. **(A)** Solvent control, **(B)** BAY-Sol (BAY-876 solution), and **(C)** BAY-Micro (Bay-876 microcrystalline) formulations were directly injected into the subcutaneous tumor tissues. **(A-C)** The rats were analyzed using microPET over a series time points. The results are shown as representative microPET images. **(D)** The quantitative results of the images. **(E)** Images of the liver organs with tumor lesions, and **(F)** quantitative results of the images. *P<0.05.

The above results were obtained using a subcutaneous tumor model. To further examine the antitumor activity of the Bay-876 formulations, an intrahepatic tumor model was established using immunodeficient rat to mimic the intrahepatic lesions of HCC. The inhibitory activity of Bay-876 formulations was also directly measured by the microPET scans at each time point. As shown in **Figure 6**, after a single injection of BAY-876 solution or microcrystalline BAY-876 into the intrahepatic lesions formed by MHCC97-H cells in the model rats’ liver organs, the BAY-876 microcrystalline-treated group showed a slower onset (3rd day time point), but it continued to inhibit the uptake of 18F-FDG for a prolonged period of time (3–20 days). However, the intrahepatic injection of the BAY-876 solution could only inhibit the uptake of 18F-FDG by over a short time (at the 24-hour time point) after injection. There was no effect of solvent control on 18F-FDG uptake.

As shown in **Figures 6E, F**, 25 days after administration, the tumor size (the total area of lesions/liver organs) of the control group and the BAY-876 solution treatment groups were similar. In contrast, the size of the intrahepatic lesions in the microcrystalline BAY-876-treated group (BAY-Micro) were much smaller than those of the control group and the BAY-876 solution group. As shown in **Table 3**, the one dose of intra-tumor injection of BAY-876 solution or BAY-876 microcrystalline formulation (BAY-Micro) into the intrahepatic lesions formed by MHCC97-H did not affect the body-weights or liver-weights of immunodeficient rat mentioned in **Figure 6**. Therefore, the intrahepatic injection of the microcrystalline BAY-876 formulation achieved the long-term inhibition of HCC proliferation and glucose uptake, and did not induce the significant toxicity, e.g. body-weights loss in animal or the significant injury of the adjacent liver tissue.

**Table 3:** The body-weights and liver-weights of rats mentioned in **Figure 6**.

Groups	Body weight (g)	Liver (g)
Untreatment	295.16 ± 18.02	3.20 ± 0.71
Solvent Control	285.91 ± 34.77	3.15 ± 0.61
BAY-Sol	289.10 ± 38.55	3.13 ± 0.45
BAY-Micro	290.99 ± 40.86	3.17 ± 0.43

BAY-Sol, BYA-876 solution; BAY-Micro, BAY-876 microcrystalline formulation.

## Discussion

Despite the efforts of the public health system and the disease prevention strategies and controls, the diagnosis and treatment of advanced HCC in China is currently facing enormous challenges (48–50). For advanced HCC lesions with clear vascular invasion, embolization or chemoembolization can be performed through the hepatic artery or portal vein (51–53). However, advanced HCC is extremely insensitive to cytotoxic chemotherapeutic agents, and the overall prognosis of patients receiving molecular targeted therapies is still not satisfactory (53). Currently, the first-line therapeutic strategy for advanced HCC is that of molecular targeted agents, of which sorafenib is the main representative. Sorafenib can directly inhibit activation of Mitogen-activated protein kinase (MAPK) signaling pathway or the activity of tyrosine kinase receptors to inhibit the angiogenesis, metastasis, and the proliferation of HCC cells. However, the toxic adverse effects of existing molecular targeted agents cannot be ignored, and furthermore, patients are also prone to resistance to these agents (28, 54–57). Thus, it is necessary to clarify the molecular mechanism of resistance or insensitivity to existing drugs. Conversely, it is necessary to discover novel therapeutic targets or strategies. In order to overcome these obstacles, treatment with BAY-876, an inhibitor of the GLUT1 receptor was investigated taking advantage of a novel microcrystalline BAY-876 formulation. BAY-876 is a synthetic and verified antagonist for GLUT with good inhibitory activity against GLUT1 (the IC_50_ value of 2 nmol/L against the GLUT1 receptor in cell-free system) (29). However, the chemical properties of BAY-876 indicated that BAY-876 could not be solubilized in water. This makes conventional treatment strategies such as oral or intravenous injection of BAY-876 very difficult. A common research strategy is to prepare the drug as a sulfate, hydrochloride, or besylate derivative to improve the solubility of the drug. Our considered another standpoint, using the water-insoluble property of BAY-876, we prepared a microcrystalline BAY-876 formulation with a relatively uniform particle size. The BAY-876 microcrystalline formulation was directly injected into the tumor tissue. As the HCC tissue gradually dissolves the BAY-876 microcrystalline formulation, the drug is slowly released. This can not only achieve a sustained release effect of BAY-876 in localized to HCC tissues, but also a single administration (ie a single dose of intratumor injection) can achieve long-term anti-tumor effects. This approach not only expands our understanding of BAY-876 antitumor activity, but also provides a new strategy for research of water insoluble drugs.

Moreover, an aberrant metabolism characterized by impaired glucose uptake or by aerobic glycolysis is associated with the overexpression of GLUT1 (58–63). The aberrant metabolism of HCC cells also participates in the progression of HCC by influencing the tumor microenvironment (14, 64–67). Thus, GLUT1 is a promising therapeutic target for HCC. Conversely, GLUT1 activity is also essential for normal tissues (such as the brain, internal organs, and muscles) for glucose uptake and for normal physiological activities. Oral administration of BAY-876 or intravenous injection of BAY-876 will lead to the systemic distribution of BAY-876 throughout the body, which seriously interferes with the physiological activity of normal tissues. The BAY-876 microcrystalline formulation prepared in this study not only achieves the sustained release and long-term anti-tumor activity of BAY-876 in HCC tissues, but it also achieves a localized administration of BAY-876. Our findings indicate that after direct injection of the microcrystalline BAY-876 formulation into tumor tissues, the concentration of the drug in the blood of nude mice remained at extremely low levels, which avoided the potential toxicity and side effects caused by BAY-876. Similarly, the anti-tumor activity of BAY-876 was reflected by changes in tumor volume and tumor weight, but were also reflect by direct effects of BAY-876 on HCC tissue metabolism through the changes in lactate, LDH, ATP, and glucose uptake. In addition, the detection of the expression of EMT related factors in HCC tissue was also influenced by BAY-876 activity.

Moreover, other reports have described that GLUT1 activity is regulated by different mechanisms: (1) the PI3K/AKT/mTOR pathway (65) participates in the regulation of tumor metabolism by GLUT1, and interference with the PI3K/AKT/mTOR pathway can effectively inhibit the expression of GLUT1 (68); (2) using small interfering RNA to down-regulate the expression of GLUT1, cell proliferation is significantly inhibited (69); (3) glucose starvation can induce an increase in the expression level of GLUT1 mRNA, and this effect depends on the activation of the AMPK pathway (70); and (4) oncogenes/proto-oncogenes, such as KRAS and BRAF mutations (71), can promote the expression of GLUT1, which directly increases the uptake of glucose by tumor cells (70, 71). The above findings indicate that the combined use of BAY-876 and inhibitors of the above-mentioned mechanisms/pathways may also be of great significance. Similar to our approach on directly inhibiting the activity of GLUT1 in HCC using BAY-876, Ma et al. used a microRNA (miR-6077) to directly down-regulate the expression of GLUT1, to achieve anti-tumor effects.

For now, molecularly targeted drugs are still the main treatment strategy for advanced HCC (72–74). Among them, Sorafenib is the most widely used (75–77). In order to overcome the shortcomings of Sorafenib, newly molecular targeted drugs including Regorafenib, Lenvatinib and Cabozantinib have been approved for marketing one after another (78–80). Although it is currently believed that these drugs are generally superior to Sorafenib, these agents (lenvatinib, regorafenib, and carbozantinib) have the same chemical core structure (1-(4- (pyridin-4-yloxy)phenyl) urea) with sorafenib (81). Therefore, it is urgent to develop the novel research strategies. In this study, the clinical significance of GLUT1 expression in HCC was first revealed, and then a novel and more effective treatment strategy for BAY-876 was established. The BAY-876 related research in HCC is of great significance and very reasonable. The liver is the center of human material and energy metabolism and the most important organ for glucose metabolism. For glucose metabolism, the liver is the center of hepatic glycogen synthesis and gluconeogenesis, and glucose uptake mediated by GLUT1 is essential for this process (82–89). At the same time, HCC is derived from normal liver cells, and HCC not only has more vigorous material and energy metabolism, but also has the characteristics of aerobic glycolysis and Warburg (47). All these make HCC, BAY-876 and GLUT1 related research of great significance. Increasing evidence have indicated that the GLUT1 could function as a positive regulator of HCC’s proliferation or metastasis (90–93). Li et al., 2021 indicated that the HCC has the high uptake of [(18)F]FDG feature and Xia et al., 2020 revealed that the glucose transporter represented by GLUT1 impacted (18)F-fluorodeoxyglucose PET-CT imaging in hepatocellular carcinoma (94, 95). Results from Yi et al., 2020 showed that repressing of GLUT1 *via* miR-328-3p not only exert antiproliferative activation but also enhanced the sensitivity of cells to chemotherapeutics (96). Moreover, BAY-876 also showed the anti-tumor activity in ovarian cancer model (97), which indicates the importance of the selection of cancer type (97). Therefore, the selection of cancer type may depend on whether the expression of GLUT1 is positive.

The oral administration of drug are adapted to the type of chronic diseases that require long-term medication such as diabetes (98–100). Human malignacies are also considered to be chronic diseases, however, the recent research strategy of anti-tumor drug treatment has always been to try to achieve long-term oral anti-tumor drugs or to extend the cycle or time interval of intravenous drugs as much as possible (101–103). For example, albumin paclitaxel has been widely used clinically, and it can be injected once a week or a month in the treatment of breast cancer or lung cancer (104, 105). Nevertheless, due to the high toxicity of anti-tumor drugs themselves, if oral administration or intravenous injection will cause the drug to be distributed throughout the body, on the one hand, other organs will be damaged, and on the other hand, the concentration of the drug in the lesion will be limited. Various interventional treatment strategies represented by transhepatic arterial chemoembolization (TACE) aim to achieve precise administration of HCC anti-tumor therapy (49, 106). DSA-guided transhepatic arterial chemoembolization or CT-guided percutaneous puncture can realize the direct administration of drugs to tumor tissues (107–110). At the same time, the most commonly used and latest technology is various drug sustained-release microspheres, which can achieve long-acting and sustained release of drugs in tumor tissues (111–114). The agents were physically adsorbed to the microspheres. As an example, the results from Shi et al., 2020 shown that prepared the Apatinib as loaded CalliSpheres Beads for embolization could achieved the good pharmacokinetics feature and tumor response in liver tumor model (115). However, the usage of the microspheres still has some shortcomings: the microspheres such as CalliSpheres Beads were made by the polymer materials and these polymer materials may not be biodegradable. The drug-loading microspheres are mainly the microspheres themselves and amount of drug-loaded by microspheres is limited. This makes the microcrystalline prepared in this study have corresponding advantages: it did not contain polymer materials and can achieve a dosage of more than 30mg/ml of BAYA-876. The particle size of BAY-876 microcrystalline can also be controlled to realize the TACE of BAY-876. At the same time, the preparation of BAY-876 microcrystals with a larger particle size can not only play an anti-tumor effect, but may also play a role in embolizing tumor micro-vessels. For translation of BAY-876 microcrystals in the clinic, transhepatic artery chemoembolization or incisive puncture can be used, and the drug can even be prepared as an implant (116, 117).

## Conclusion

This study first clarified the relationship between the expression of GLUT1 and the prognosis of patients with advanced HCC receiving Sorafenib treatment, and the role of GLUT1 in the proliferation of HCC cells. On this basis, BAY-876 microcrystals were prepared, realizing the long-acting and precise administration of BAY-876. This research not only expands our understanding of HCC-GLUT related research, but also helps to provide new enlightenment for HCC treatment.

## Data Availability Statement

The original contributions presented in the study are included in the article/**Supplementary Material**. Further inquiries can be directed to the corresponding authors.

## Ethics Statement

The animal study was reviewed and approved by Animal Ethics Committee of Affiliated Hospital of Hebei University.

## Author Contributions

HY, MZ, and H-wS: concept, design, statistics, data collection, manuscript writing, final approval. JH and QJ: design, statistics, data collection. H-wS: concept, data collection. Y-tC, X-jL, JH, and QJ: statistics, manuscript writing. HY: statistics, data collection. HY, QJ, and JH: statistics, data collection. HY, QJ, and JH: concept, design, statistics, data collection, manuscript writing, final approval. All authors contributed to the article and approved the submitted version.

## Conflict of Interest

The authors declare that the research was conducted in the absence of any commercial or financial relationships that could be construed as a potential conflict of interest.

## Publisher’s Note

All claims expressed in this article are solely those of the authors and do not necessarily represent those of their affiliated organizations, or those of the publisher, the editors and the reviewers. Any product that may be evaluated in this article, or claim that may be made by its manufacturer, is not guaranteed or endorsed by the publisher.
