# Supplementary material for: A Novel Microcrystalline BAY-876 Formulation Achieves Long-Acting Antitumor Activity Against Aerobic Glycolysis and Proliferation of Hepatocellular Carcinoma
Source: Front Oncol. 2021 Nov 18;11:783194. doi: 10.3389/fonc.2021.783194 (PMC8636331; doi:10.3389/fonc.2021.783194)
Supplement: Supplementary file 1 [file DataSheet_1.doc]

Supplemental Table 1 the Reverse transcription experiment system settings

| Agents | Volumes |
| --- | --- |
| 50ng/ul random hexamers | 1 μl |
| 10mM dNTP mix (10mM each) | 1 μl |
| Total RNA | 11μl |

Supplemental Table 2 Reverse transcription experiment system settings

| Agents | Volumes |
| --- | --- |
| the above mentioned reagents | 13 μl |
| 5×SSIV Buffer | 4 μl |
| 100 mM DTTL | 1 μl |
| Ribonuclease Inhibitor | 1 μl |
| SuperScript™ IV Reverse Transcriptase (200 U/uL) | 1 μl |

Supplemental Table 3 Setting of Quantitative PCR Reaction System

| Agents | Volumes |
| --- | --- |
| KAPA SYBR FAST qPCR Master Mix (2X) Universal | 10 μl |
| 10mM dUTP | 0.4 μl |
| 10μM forward primer | 0.4 μl |
| 10μM reverse primer | 0.4 μl |
| 50X KAPA RT Mix | 0.4 μl |
| Template DNA | 1.5 μl |
| 50X ROX Low | 0.4 μl |
| PCR-grade water | 6.5 μl |

**Supplemental Table 4 The HPLC mobile phase conditions**

| Time (min) | B liquid ratio (%) |
| --- | --- |
| 0 | 2 |
| 4 | 80 |
| 4.1 | 100 |
| 6 | 100 |
| 6.1 | 2 |
| 10 | 2 |
